# Examining the Relationships Between Parenting Practices, Children’s Temperament, and Academic and Behavioural Outcomes in Lower-Income Families

**DOI:** 10.3390/bs15060786

**Published:** 2025-06-06

**Authors:** Calpanaa Jegatheeswaran, Samantha Burns, Michal Perlman

**Affiliations:** Applied Psychology and Human Development, University of Toronto, Toronto, ON M5S 1V6, Canada; calpanaa.jegatheeswaran@mail.utoronto.ca (C.J.); samantha.burns@utoronto.ca (S.B.)

**Keywords:** parenting, temperament, structural equation modelling, child outcomes, low-income

## Abstract

Maternal childrearing practices play a prominent role in a child’s developmental outcomes. Difficult child temperament, specifically, negative emotionality, impacts parenting practices. The present study contributes to the existing literature by investigating the mediating role of parenting practices on associations between children’s temperament and academic and behavioural outcomes in a low-income and ethnically diverse sample. The present study consists of a sample of 163 families. The average age of the children was 32.40 months (SD = 2.61 months). The average age of the mothers was 34.35 years (SD = 5.32 years). Structural equation modelling examined the relationship between children’s temperament, parenting practices, and child outcomes. A two-step procedure was conducted to test this model: confirmatory factor analysis followed by latent path analysis. The results show that children’s temperament was significantly and positively associated with mothers’ hostile parenting and children’s conduct problems. Hostile parenting was positively associated with children’s conduct problems. While overprotective parenting was negatively associated with children’s receptive vocabulary scores, maternal responsivity was positively associated with better receptive vocabulary in children. Finally, hostile parenting was found to play a significant and positive mediating role in children’s conduct behaviour. Maternal practices are associated with outcomes in children with negative emotionality, underscoring the need for tailored interventions in diverse, low-income families.

## 1. Introduction

The parent–child relationship influences children’s developmental trajectories (Giannotta & Rydell, 2016), with parents being key socializing agents in their children’s lives (Bandura, 1977; Morris et al., 2002). Accordingly, the quality of these relationships can positively or negatively impact children’s outcomes, including their socio-emotional and cognitive well-being (Madigan et al., 2019; Pinquart, 2017).

### 1.1. Understanding Parenting Practices

Parenting practices are observable and are defined as routine childrearing behaviours parents engage in when interacting with their children (Bornstein et al., 2011). Positive parenting practices contribute to forming a secure parent–child attachment and lead to favourable child outcomes (e.g., pre-academic skills; Wade et al., 2018). For instance, responsive parenting practices support children’s socio-emotional and cognitive development (Claussen & Crittenden, 2000; Madigan et al., 2019). In line with attachment theory (Ainsworth, 1978), emotional responsivity is characterized by sensitivity, warmth, and affection when responding to children, especially in times of distress. As per Vygotsky’s (1934/1986) sociocultural theory, cognitive responsivity encompasses prompt and developmentally appropriate responses to exploratory and communicative interactions with children (i.e., providing rich verbal input and scaffolding). Responsive parents tend to have highly engaging conversations with their children (e.g., labelling objects and describing their attributes). Children of responsive parents often demonstrate early academic skill development (i.e., reading, verbal ability, and math; Hart & Risley, 1996; Wade et al., 2018) and socio-emotional skills (Bakermans-Kranenburg et al., 2003).

Dysfunctional parenting practices on the other hand contribute to insecure attachment with children and negative child outcomes such as internalizing and externalizing symptoms (Clayborne et al., 2021; Pinquart, 2017). Examples of dysfunctional parenting practices include parental overprotection and hostility. Overprotective parenting consists of “excessive parental regulation of children’s activities and routines, encouragement of children’s dependence on parents, and instruction to children on how to think or feel” (McLeod et al., 2007, p. 156). Empirical evidence has repeatedly demonstrated the negative relationship between parental overprotection and children’s outcomes, including avoidant coping and anxiety symptoms (Borelli et al., 2015) and hyperactivity–impulsivity and behavioural symptoms (Faleschini et al., 2020; Gere et al., 2012). Overprotective parents also tend to excessively and deliberately intervene in their children’s learning experiences (in a loving but misguided manner), removing obstacles or challenges that may arise, often by stepping in and solving problems for their children (Locke et al., 2012). For instance, a study by Valdez (2016) found that parental overprotection was negatively associated with academic self-esteem in preschool-aged children. Hostile parenting is characterized as “overt verbal and physical aggression toward the child” (Morris et al., 2002, p. 462). Many studies have found that hostile parenting behaviours lead to poorer outcomes in children, including externalizing problems (aggression and conduct problems; Morris et al., 2002), negative emotionality and delinquency (Braungart-Rieker et al., 2010), and hindered executive functioning (Lam et al., 2018). In addition, insensitive maternal responses (i.e., mothers who are hostile and dismissive of their children’s needs and cues) can lead to maladaptive self-regulation skills in children (Feldman et al., 2009).

### 1.2. The Influence of Child Temperament

It is important to note that the parent–child relationship is not unidirectional since children’s innate characteristics can influence their environment (i.e., gene–environment correlation; Plomin et al., 1977). In 1984, child psychologist Jay Belsky put forth *The Determinants of Parenting: A Process Model* (Belsky, 1984), which recognizes child characteristics (e.g., child temperament) as important determinants of parenting. Biologically based variances in children’s emotional, motor, and attentional reactivity, as well as their patterns of self-regulation, are referred to as child temperament (Putnam et al., 2002). The literature has extensively examined the association between difficult temperament, specifically negative emotionality (e.g., irritability, anger, fear, and/or sadness), and child behavioural and academic outcomes (Eisenberg et al., 2010; Noel et al., 2008). For instance, an angry temperament has repeatedly been shown to be more related to externalizing problems in children (Kostyrka-Allchorne et al., 2020).

### 1.3. Child Temperament, Parenting Practices, and Mediation Pathways

Given the biological properties of temperament, it is viewed as a risk factor for poor parenting quality. The child’s temperament influences the parent’s behaviour (Lozano et al., 2020), as children who habitually exhibit difficult child temperament are perceived as harder to manage by their parents (Crockenberg & Leerkes, 2003). Research has consistently shown that parents whose children exhibit negative emotionality tend to use harsher and more hostile parenting methods to discipline, control, or manage them (Laukkanen et al., 2014; Paulussen-Hoogeboom et al., 2007; Shewark et al., 2021; Taraban & Shaw, 2018). A meta-analysis by Paulussen-Hoogeboom et al. (2007) found a small but significant effect on the inverse association between negative emotionality in children and supportive parenting. In addition, the meta-analysis demonstrated a small significant effect between child negative emotionality and higher restrictive control (i.e., overprotection).

Considering the relationship between child temperament and parenting practices, along with the impact of these practices on children’s developmental trajectories, it is reasonable to hypothesize that the link between temperament and outcomes is partially mediated by children’s experiences with their parents. Various studies have explored this mediation pathway (Lozano et al., 2020; Olson et al., 2005; Paulussen-Hoogeboom et al., 2008; Prinzie et al., 2010). As an example, Paulussen-Hoogeboom et al. (2008) found that mothers’ authoritative parenting style partially mediated the relationship between negative emotionality and internalizing and externalizing behaviours in children. Similarly, a study by Shewark et al. (2021) found that child anger at four-and-a-half years evoked hostile parenting from adoptive parents at six years, which was subsequently related to child problem behaviours at seven years.

The current study seeks to introduce two novel contributions to this field of research. First, it aims to disentangle the distinct impacts of various types of parenting practices on the temperament–outcome relationship. Second, it focuses on understanding these associations within the context of diverse, low-income families, a demographic often underrepresented in previous research (Paulussen-Hoogeboom et al., 2008). Parenting practices are central to child development, but their influence may be particularly pronounced in lower-income families, where environmental stressors such as financial instability, limited access to resources, and heightened parental stress can negatively impact parenting quality. Prior research has demonstrated that these stressors can lead to harsher parenting practices, which, in turn, are associated with poorer child outcomes (Cybele raver, 2003; Rafferty & Griffin, 2010). Despite this, the role of parenting as a mediator between difficult child temperament (which is associated with poorer parenting; Paulussen-Hoogeboom et al., 2007) and developmental outcomes in lower-income families remains relatively underexplored. The present study addresses this gap by examining how mothers’ parenting practices mediate the relationship between children’s temperament and their academic and behavioural outcomes in a sample of low-income families.

### 1.4. Present Study

The present study empirically investigates the mediating role of parenting practices in the relationship between children’s temperament and academic and behavioural outcomes. Traditionally and cross-culturally, mothers assume the role of primary caregiver. Consequently, children spend significantly more time interacting with their mothers, and when the relationship is positive, they perceive their mothers as a source of comfort and security (Hartup, 1989; Milkie et al., 2015). The impacts of mothers’ childrearing practices have been well-established and are understood to play a prominent role in children’s development outcomes (Jegatheeswaran et al., 2023). Therefore, the present study will focus on the mother–child relationship.

We aim to address the following research questions:

1. Is there an association between maternal reports of children’s negative temperament (i.e., greater anger/frustration) and the use of negative parenting practices (i.e., lower maternal responsivity and higher levels of overprotectiveness and hostility)?
  We hypothesize that mothers who report that their children display greater levels of anger and frustration are more likely to utilize negative parenting practices.
2. Is there an association between negative parenting practices and child academic and behavioural outcomes?
  We hypothesize that negative parenting practices—including lower maternal responsivity and higher levels of overprotection and hostility—are associated with poor academic and behavioural outcomes in children.
3. Do parenting practices mediate the relationship between children’s temperament and academic and behavioural outcomes?
  We hypothesize that negative parenting practices may help explain the observed associations between children’s negative temperament and poorer academic performance and behavioural outcomes.

## 2. Materials and Methods

### 2.1. Participants

The present study draws on data from *Child Care Matters*, which is a larger longitudinal study. Psychological and environmental data were collected via telephone and in person across multiple waves of data collection, starting when children were 12 months old. Recruitment took place between 2014 and 2016. The original sample consisted of 895 low-income families from the City of Toronto’s childcare subsidy. This study used data collected approximately 2 years later, when children were between the ages of 30 and 42 months. Families would have had to meet two selection criteria to be included in the present study. Firstly, mothers (as opposed to fathers) would have had to have completed the in-person survey. Secondly, in addition to the survey, mother–child dyads would have completed an in-person task assessing maternal responsivity and children’s receptive vocabulary skills (described below). This in-person assessment took place in one of the city’s four regional offices and was based on the convenience of the individual mothers. After applying the selection criteria, we were left with 163 families. A power analysis determined that a sample size of 137 participants was required to detect a moderate effect size of 0.3 or higher with 80% statistical power (Soper, 2025).

Approximately half (50.90%) of the children were males. The average age of the children was 32.40 months (range = 30 to 42 months; SD = 2.61 months). The average age of the mothers was 34.35 years (range = 23 to 49 years; SD = 5.32 years). Most of our sample came from a two-parent home (65.10%). The average income of the households in the sample was between CAD 50,000 and CAD 59,999. This is significantly lower than the average household income in Toronto of CAD 104,378 (City of Toronto, 2017). Finally, a total of 18.40% of mothers had less than a college certificate, most had a college diploma or some university attendance (35.60%), some had a bachelor’s degree (28.80%), and few had a master’s degree or above (17.20%).

Ethics approval for the larger study was obtained through the University of Toronto Research Ethics Board. The study was approved to be conducted within communities in the Greater Toronto Area.

### 2.2. Measures

#### 2.2.1. Demographics

The ages of the children and their mothers were collected in months and years, respectively. To assess family income, mothers were asked, “What was your total family income before taxes last year?” Our sample consisted of low-income families. As described above, the average income of the households in the sample was between CAD 50,000 and CAD 59,999, significantly lower than the average household income in Toronto of CAD 104,378 (City of Toronto, 2017). For data analysis, we categorized families within our lower-income sample into two groups: relatively lower-income (i.e., ‘CAD 59,999 and below’) and relatively higher-income (i.e., ‘CAD 60,000 and above’). Lastly, to assess educational background, mothers were asked, “What is the highest level of education you have completed?” The responses were dichotomized for data analysis into ‘College and below’ and ‘above College.’

#### 2.2.2. Child Temperament

Early Childhood Behaviour Questionnaire (ECBQ)—Short Form. The ECBQ is a questionnaire completed by parents to evaluate temperament in toddlers aged 1 to 3 years (Putnam et al., 2006). The ECBQ Short Form includes 107 questions that contribute to 18 subscales. The present study utilized six items that load on the anger/frustration subscale (e.g., “negative affect related to interruption of ongoing tasks or goal blocking”; Putnam et al., 2010). Parents rated their child’s anger/frustration frequency over the previous two weeks using a 7-point Likert scale, ranging from “never” to “always”. The mean score was calculated for the relevant items. The ECBQ shows high internal consistency for the anger/frustration dimension (α = 0.73 for each) and concurrent validity with the standard ECBQ (*r* = 0.75, respectively; Putnam et al., 2010).

Child Behaviour Questionnaire (CBQ)—Short Form. In this study, some children surpassed the age limit for the ECBQ, so the anger/frustration subscale from the CBQ Short Form, which consists of six items, was used in those instances. The CBQ is a parent-reported questionnaire designed to evaluate temperament in children aged 3 to 7 years (Rothbart et al., 2001). Parents rate their child’s anger/frustration over the past six months using a 7-point Likert scale, ranging from 1 (“extremely untrue of your child”) to 7 (“extremely true of your child”). Scores were calculated by averaging the relevant items for each child (Putnam et al., 2006). The anger/frustration dimension presented adequate internal consistency (α = 0.69–0.78; Putnam et al., 2006), showed high correlations with the standard form CBQ (*r* = 0.75; Putnam et al., 2006), and was found to be a valid and reliable measure of temperament in pre-school years (de la Osa et al., 2013).

#### 2.2.3. Parenting

Responsive Interactions for Learning (RIFL). The RIFL measure (Prime et al., 2014, 2015) was used to assess the capability of mothers to respond to their children’s cognitive needs when engaged in a goal-oriented activity. Mother–child dyads were filmed for 5 min, during which they engaged in a cooperative building task using Duplo blocks. Raters watched and coded the 5 min clips based on general impressions using an 11-item, 5-point Likert scale, ranging from 1 (“Not at all true”) to 5 (“Very true”). Inter-rater reliability was established between an expert and each of the five coders who coded the videotapes for this study. Total scores were determined using Cronbach’s alpha, grounded in generalizability theory (Cronbach et al., 1972), along with the guidelines provided by Stemler (2004). Inter-rater reliability across the five coders was high (α = 0.84).

This scale comprised three conceptual dimensions: communicative clarity (6 items), mind-reading (3 items), and mutuality building (2 items). A higher average score indicates higher levels of maternal responsivity. Prime et al. (2015) confirmed the 1-factor structure of this measure using confirmatory factor analysis. The RIFL measure demonstrates excellent internal consistency (α = 0.92) and functions similarly to standardized maternal responsivity measures while demonstrating divergent validity (Prime et al., 2015).

The Parental Cognitions and Conduct Toward the Infant Scale (PACOTIS). The PACOTIS is a 23-item parental self-report that measures parental perceptions and behaviours towards their infant (Boivin et al., 2005). Scores encompass a rating on a Likert-type scale ranging from 0 (“not at all what I think”) to 10 (“exactly what I think”), with higher scores indicating stronger behaviours. Our study will utilize the parental hostile-reactive behaviours and overprotection subscales. The PACOTIS has high internal consistency (Cronbach’s α = 0.75; Črnčec et al., 2010).

#### 2.2.4. Child Outcomes

Peabody Picture Vocabulary Test Fourth Edition (PPVT-IV). The PPVT-IV is an untimed receptive vocabulary test for children aged two years or older, consisting of 228 items arranged to increase difficulty (Dunn & Dunn, 2007). The experimenter pronounces a word, and participants select from an array of four pictures the one that best describes the word; the test is terminated when the participant provides eight incorrect responses out of a set of 12 (Kasari et al., 2014). The test provides a standardized receptive vocabulary score based on a normed sample with a mean standard score of 100 and a standard deviation of 15 (Spaulding et al., 2013). The PPVT-IV has been found to show good internal consistency, test–retest reliability and concurrent validity (Besha et al., 2017). Evidence for the reliability of PPVT–IV scores included a mean alpha coefficient of 0.97 and a mean test–retest reliability coefficient of 0.92 (Nelson & Canivez, 2012).

The Strengths and Difficulties Questionnaire (SDQ; Goodman, 1997; Youth in Mind, 2014). The SDQ is a 25-item parent-report questionnaire to capture children’s internalizing and externalizing problems. Participants rated the items on a 3-point Likert scale as either “Not true” (0), “Somewhat true” (1), or “Certainly true” (2). The present study used only the conduct problem to address the initial research question. The SDQ has high discriminant validity across the five subscales (Croft et al., 2015) and demonstrates satisfactory internal consistency (α > 0.70; D’Souza et al., 2016).

### 2.3. Data Analysis

All data analyses were computed using MPlus (Muthén & Muthén, 2017). The research questions of interest include both latent constructs and correlational analyses; therefore, structural equation modelling (SEM) was chosen. SEM allows for the measurement and examination of the study’s latent constructs (i.e., parenting practices) instead of simply creating manifest scores (Kline, 2010). In addition, SEM can account for measurement error when examining the relationship between the latent construct and multiple variables, as well as examining both direct and indirect (i.e., mediation) effects. A two-step procedure was conducted to test this model (Anderson & Gerbing, 1988): the Measurement and Structural Models.

#### 2.3.1. Building the Measurement Model

According to Anderson and Gerbing (1988), the first step is to evaluate the measurement model prior to analyzing the structural regression paths. Therefore, the measurement model was specified and estimated by conducting a confirmatory factor analysis (CFA; Appendix A).

Due to the complex nature of the variables included in the structural equation model, analyses began with four separate measurement models (i.e., conduct problems, hostile parenting, overprotection, and responsivity). Two of the four produced good-fitting models that ran as expected and did not require modifications. Responsivity had one index that was slightly higher than the 0.08 RMSEA cut-off. However, conduct problems failed to meet model fit criteria across a range of indices. Thus, modifications were conducted and the model ran appropriately. The respecified measurements were then included in one model, which also demonstrated a good model fit.

#### 2.3.2. Building the Structural Model

The second step included latent path analysis techniques, which include a mediation model.

The structural model included the measurement models described above. Additionally, the structural model estimated the associations among the constructs. Specifically, associations were estimated between child temperament and parenting (i.e., responsivity, overprotection, and hostile parenting). Associations were also specified between the parenting constructs and child outcomes (i.e., receptive vocabulary and conduct problems). Covariates in the model include total family income, maternal education, and the ages of the mother and child. Finally, shared variance across the three parenting constructs was accounted for.

A child’s temperament was expected to have a relationship with their academic and behavioural outcomes, and an indirect association mediated by parenting practices. Model fit indices were used to determine how well the measurement and structural models fit the data. Hu and Bentler (1999) provide a review of interpreting model fit indices. The first is the X^2^ goodness-of-fit statistics, which assesses the magnitude of discrepancy between the fitted matrices and the sample. The second model fit index is complementary to the X^2^ test, which assesses the degree to which an a priori model reproduces the data. A X^2^ *p*-value cut-off less than 0.05 will be implemented. The absolute fit indices cut-off we elected to use is the Root Mean Square Error of Approximation (RMSEA; Steiger & Lind, 1980) cut off 0.05 to 0.08, as they are considered indicative of fair fit, and values greater than 0.10 poor fit (Browne & Cudeck, 1993), and 0.80 to 0.10 are indicative of mediocre fit (MacCallum et al., 1996). The incremental fit indexes include the Tucker–Lewis Index (TLI; Tucker & Lewis, 1973) and Bender’s Comparative Fit Index (CFI; 1990) with a cut-off of 0.90.

## 3. Results

### 3.1. Descriptive Analyses

The mean and standard deviations for all variables used in this study are reported in Table 1. Associations between each of the covariates and latent constructs are reported in Table 2. Only two latent constructs had significant associations with the covariates. Specifically, there was an association between income and overprotective parenting; lower total family income (defined as below CAD 59,999) was related to higher scores on overprotective parenting. Furthermore, higher income (above CAD 60,000) and education (above a college diploma) was associated with higher responsiveness when interacting with their children.

### 3.2. Child Temperament on Parenting Practices and Child Outcomes

**Figure 1:**
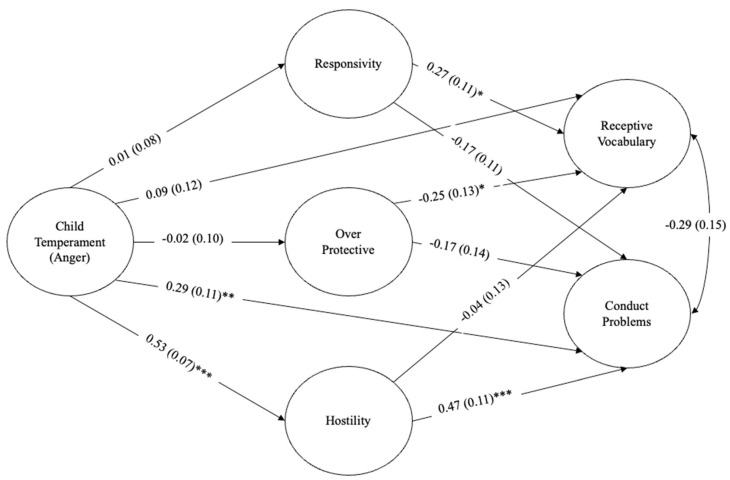
Structural path model with standardized estimates (standard errors in parentheses). The covariates (i.e., child- and parental-level variables) are not shown in the figure for clarity. Information regarding the relationships between covariates and constructs can be seen in Table 2. * *p* < 0.05, ** *p* < 0.01, *** *p* < 0.001.

The structural path analysis and its associated estimates are presented in Figure 1. Measurement and structural model fit indices can be found in Table 3. There was a positive and significant relationship between child temperament and hostile parenting (β = 0.53, *p* ≤ 0.001). These findings indicate that mothers were more hostile when their children had more negative temperaments. This relationship remained significant even when controlling for the age of the mother and child, total family income, and the mother’s education level. Children’s temperaments had no significant relationship with any other parenting constructs. Regarding children’s outcomes, there was a positive and significant relationship between temperament and conduct problems (β = 0.29, *p* ≤ 0.01). That is, children had more conduct problems when they were described as having more angry temperaments.

### 3.3. Parenting Practices on Child Outcomes

Higher levels of hostile parenting was significantly associated with increased levels of conduct problems in children (β = 0.47, *p* < 0.001). Maternal responsivity and overprotection were not significantly associated with children’s conduct problems. Children’s receptive vocabulary was significantly associated with maternal responsivity (β = 0.27, *p* ≤ 0.05) and overprotectiveness (β = −0.25, *p* ≤ 0.05). Specifically, children whose mothers were more responsive and less overprotective had better receptive vocabulary. The relationships between the three parenting constructs were not significant, except for a significant negative association between responsivity and overprotection (r = −0.26, *p* = 0.018). This finding indicates that mothers who were more responsive to their children also exhibited less overprotective behaviours. Finally, the associations between receptive language and conduct problems were not significant (r = −0.29, *p* = 0.052).

### 3.4. Mediating Role of Parenting Practices

The indirect associations between child temperament and academic and behavioural outcomes through parenting practices were examined. Specifically, associations between temperament and conduct problems were examined with responsivity (β = −0.002, SE = 0.01, *p* = 0.905), overprotectiveness (β = −0.003, SE = 0.02, *p* = 0.879), and hostile parenting (β = 0.25, SE = 0.07, *p* < 0.001) tested as potential mediators. The results suggest that hostile parenting behaviours are significantly associated with the link between a child’s temperament and their conduct problems. Specifically, lower levels of hostile parenting were related to a weaker association between children’s angry and frustrated temperament and conduct-related behavioural problems. No significant relationships were found between temperament and receptive vocabulary through parenting constructs.

## 4. Discussion

The current study examined associations between children’s temperament and mothers’ parenting, as well as the associations between parenting and children’s academic and behavioural outcomes, while accounting for sociodemographic factors. In addressing the first research question, mothers who reported that their children exhibited higher levels of anger were more likely to report engaging in hostile parenting practices. This aligns with previous research findings, which show that children with difficult temperaments tend to elicit more harsh and hostile parenting behaviours and less warmth from their parents (Laukkanen et al., 2014; Paulussen-Hoogeboom et al., 2007; Shewark et al., 2021; Taraban & Shaw, 2018). Associations between children’s temperament and mothers’ observed responsiveness or self-reported overprotection were not significant. This may reflect variability in mothers’ responses to difficult child temperament. Some mothers may become more responsive or intentionally avoid overprotection (i.e., attempting to soothe or guide the child without controlling their actions) to foster their child’s independence and self-regulation. Others may become less responsive and/or more controlling (Kiff et al., 2011). In addition, the way mothers respond to their child’s difficult temperament may depend on their stress levels, which are often compromised in low-income populations (Fang et al., 2024). These relationships should be further explored within this context.

The second research question examined how mothers’ negative parenting practices, particularly hostility and overprotection, as well as their responsiveness, are related to children’s academic and behavioural outcomes. The study found that greater maternal responsivity during observed interactions was associated with higher receptive vocabulary scores in children. Parental responsivity is characterized by being more attuned to children’s wants and needs (Claussen & Crittenden, 2000). Parents demonstrate responsivity when fostering supportive learning environments, responding to their children’s efforts and successes with warmth and praise, and addressing failures with empathy and learning opportunities. For example, activities like reading with children can include labelling objects or taking walks and vividly describing the surroundings (Hart & Risley, 1996). Such interactive conversations allow children to learn new words, boosting their receptive vocabulary.

Secondly, higher levels of maternal overprotectiveness were associated with lower receptive vocabulary scores in children. While responsive parents are cognizant of their children’s zone of proximal development, overprotective mothers tend to deliberately (and excessively) intervene in their children’s learning experiences (Locke et al., 2012). For example, when reading with the child and encountering an unfamiliar word, the parent might tell the child the “right answer” without allowing the child to sound it out first. Removing obstacles and challenges for the child restricts learning opportunities, which is why we may see lower receptive vocabulary scores.

No relationship was found between hostility and children’s receptive vocabulary. This aligns with prior research showing that hostile parenting is more strongly linked to externalizing behaviours, rather than cognitive or academic skills (Gershoff, 2002; Gershoff & Grogan-Kaylor, 2016). Language exposure and learning opportunities in the early years predominantly occur with the primary caregivers (Wade et al., 2018). While maternal responsivity and overprotectiveness directly curate these opportunities, and subsequently children’s academic outcomes, children’s behaviour tends to be influenced by a broader range of factors, including genetic predispositions (as observed in the present study) and quality of parenting. Accordingly, the only parenting practice that was related to children’s conduct problems in the current study was hostility. Specifically, higher levels of maternal hostility were associated with higher levels of conduct problems in children. Parents are arguably their children’s primary socializing agents. Per Bandura’s (1977) Social Learning Theory, children learn certain behaviours by observing their parents. Thus, hostile behaviours exhibited by the parents may, too, be mimicked by the children.

The final research question is the mediating effects of parenting on the relationship between children’s temperament and outcomes. Children’s anger was significantly and positively associated with their conduct problems. Given the robust association between temperament and conduct problems, these results are not surprising (Eisenberg et al., 2010; Kostyrka-Allchorne et al., 2020; Noel et al., 2008). The tendency for children with negative emotionality to exhibit psychological disorders, such as conduct issues, may be related to their lack of emotional and social capabilities. Maternal hostility was found to significantly, though partially, mediate the association between children’s angry temperament and their conduct problems. Specifically, children described by their mothers as having a more pronounced angry temperament tended to be associated with higher levels of maternal hostility, which in turn was linked to increased conduct problems in children. It has been previously found that negative emotionality predicts poor child outcomes when parents engage in poor parenting practices, interfering with their children (Karreman et al., 2010; Morris et al., 2002; Putnam et al., 2002). We are the first to demonstrate this in a low-income, ethnically diverse sample of mothers and young children. In part, this can be explained by the “continuum of caretaking casualty” (Sameroff & Chandler, 1975), where biologically vulnerable children only engage in disruptive behaviour when their parents fail to provide effective care, and the “coercive theory” (Patterson, 1982), which explains that negative behaviours are reinforced by parents and children.

### Limitations and Future Directions

Firstly, we faced challenges in collecting in-person data, where the observations of maternal responsivity were taken from many mothers in the original study. However, it is important to highlight that our sample was still low-income and highly diverse. This demographic is particularly challenging to recruit and retain in research. Therefore, despite the low response rate, our findings offer significant contributions for this understudied population. Another limitation is that, although we ensured our participants had sufficient English language proficiency to complete our surveys, administering measures (such as temperament, parenting, and child outcomes) in a non-native language could introduce additional cultural biases. This may complicate the interpretation of questions related to children’s developmental outcomes and parenting practices. Future research should explore how cultural factors influence participants’ understanding and responses to survey questions and how these factors may affect the overall validity and reliability of commonly used measures. Additionally, although temperament is considered a pre-existing risk factor, more research is needed on these associations’ longitudinal impacts and consistency in low-income and diverse families. For example, whether hostile parenting influences conduct problems across time and whether these associations have developmental differences in low-income and diverse samples. Furthermore, this study focused exclusively on maternal parenting practices. Future research should explore the roles of paternal figures and examine how diverse parenting dynamics shape child outcomes.

## 5. Conclusions

Our findings highlight that children’s temperament, particularly anger, was associated with more hostile parenting, which in turn was linked to behavioural difficulties such as conduct problems. In terms of academic outcomes, maternal responsivity was positively associated with children’s receptive vocabulary scores, whereas higher levels of overprotectiveness were linked to lower scores. These results emphasize the critical role that maternal parenting practices play in shaping children’s behavioural and cognitive development, particularly in children with more difficult temperaments. Moreover, the partial mediation effect of maternal hostility on the relationship between child temperament and conduct problems suggests that addressing dysfunctional parenting may help mitigate the impact of difficult temperaments.

A key implication of this study is the need for tailored interventions that target not only child temperament but also specific parenting practices, especially in families facing sociodemographic challenges. Parenting interventions provide parents with support, guidance, and resources, enhancing their skills and confidence in raising children and ultimately improving child and family well-being. These interventions offer positive and promising results across a variety of parent populations, including parents of typically developing children (Nieuwboer et al., 2013) and children with or at risk of mental and behavioural difficulties (Bywater et al., 2009). Parenting programmes that reduce hostile and overprotective behaviours while fostering responsivity may improve children’s academic and behavioural outcomes. For example, Triple P or Incredible Years focus on enhancing parental warmth, reducing harsh discipline, and increasing responsive interactions, which can benefit families of temperamentally difficult children (Nixon, 2001; Sanders et al., 2014)

Findings from the current study are particularly pertinent for low-income, diverse populations, where external stressors may exacerbate parenting challenges. Parenting interventions have been shown to be effective for families experiencing social disadvantage (McGilloway et al., 2012). Therefore, access to these evidence-based parenting programmes should be improved through community partnerships, culturally adapted materials, and flexible delivery formats (e.g., online, in-person, and hybrid) to ensure all families receive the support that they need.

## Figures and Tables

**Table 1:** Sample descriptives.

Variable	N	M	SD	Min	Max	α
**Child Level**						
Control Variables						
Age (Months)	161	32.40	2.61	30	42	-
Direct Child Assessment						
Temperament: Anger	163	3.41	1.04	1.17	6.17	0.76/0.24 *
Conduct Problems	162	2.26	1.78	0	8	0.57
Receptive Vocabulary	121	103.39	13.79	70	148	-
**Parent Level**						
Control Variables						
Age	150	34.35	5.32	23	50	-
Parenting Variables						
Responsivity	163	3.19	0.80	1.00	5.00	0.96
Overprotection	163	5.01	2.40	0.40	10.00	0.66
Hostility	162	1.79	1.32	0.00	6.29	0.71
**Applied to Both Levels**						
Control Variables						
Income	149	0.48	0.50	0.00	1.00	-
Education	163	0.50	0.50	0.00	1.00	-

Note: * For CBQ (above 3 years of age) n = 16.

**Table 2:** Covariate and latent construct associations.

	Responsivity	Overprotection	Hostility	Conduct Problems	Receptive Vocabulary
Covariates	β (SE)	β (SE)	β (SE)	β (SE)	β (SE)
Income	0.34 (0.08) ***	−0.37 (0.10) ***	−0.07 (0.09)	0.11 (0.11)	−0.10 (0.11)
Education	0.19 (0.09) *	−0.06 (0.11)	0.05 (0.09)	−0.18 (0.10)	−0.04 (0.12)
Mom’s Age	−0.14 (0.08)	0.02 (0.10)	−0.03 (0.08)	-	-
Child’s Age	-	-	-	−0.13 (0.09)	0.10 (0.11)

Note: * *p* < 0.05; *** *p* < 0.001.

**Table 3:** Model fit.

Model Fit Indices	AIC	BIC	RMSEA	SRMR	CFI	TLI
Measurement Model						
RIFL	3472.22	3574.32	0.10	0.03	0.96	0.94
Overprotective	4300.27	4346.68	0.05	0.03	0.98	0.97
Harsh Parenting	4527.72	4592.69	0.07	0.05	0.96	0.94
Conduct Problems ^+^	1274.99	1321.39	0.10	0.05	0.89	0.78
Conduct Problems ^++^	1098.07	1135.19	0.07	0.03	0.98	0.93
All Measures	13,355.06	13,624.21	0.06	0.07	0.91	0.91
Structural Model						
Total Model *	11,857.40	12,185.70	0.05	0.07	0.91	0.90

Note: ^+^ Model with “Often fights with other children”, ^++^ model with “Often fights with other children” removed as the criteria does not meet the cut-off, * the chi-square test of model fit was significant, χ^2^(461) = 620.43, *p* < 0.001.

## Data Availability

The datasets presented in this article are not readily available because of the privacy of the research participants. Requests to access the datasets should be directed to Michal Perlman.

## Abbreviations

The following abbreviations are used in this manuscript:

CBQ	Child Behaviour Questionnaire
CFA	Confirmatory Factor Analysis
CFI	Comparative Fit Index
ECBQ	Early Childhood Behaviour Questionnaire
PACOTIS	Parental Cognitions and Conduct Toward the Infant Scale
PPVT	Peabody Picture Vocabulary Test
RIFL	Responsive Interactions for Learning
RMSEA	Root Mean Square Error of Approximation
SDQ	Strengths and Difficulties Questionnaire
SEM	Structural Equation Modelling
TLI	Tucker–Lewis Index

## Appendix A

**Measures**	**Individual Factor Loadings**	**Individual Standard Estimate**	**Total Factor Lading**	**Total Standard Estimate**
**RIFL—Responsive Parenting**				
This mother gives clear and specific verbal directions.	0.76	0.06	0.82	0.03
This mother gives positive nonverbal directions.	0.73	0.06	0.82	0.03
This mother reminds her child about goals/rules of the task.	0.63	0.06	0.76	0.04
This mother will try to complete the task in a way that is sensitive to the child’s needs and desires.	0.86	0.06	0.88	0.02
This mother will try to follow the rules in a way that is sensitive to the child’s needs and desires.	0.88	0.07	0.86	0.02
This mother is clear in her requests for help.	0.79	0.06	0.87	0.02
This mother is sensitively responsive to her child’s requests for help, even those that are subtle/nonverbal.	0.79	0.06	0.82	0.03
This mother is good at rephrasing what her child does not understand.	0.69	0.05	0.82	0.03
This mother is sensitive to what her child knows and/or understands.	0.85	0.07	0.82	0.03
This mother gives positive feedback to reinforce her child.	0.67	0.07	0.68	0.04
This mother promotes turn taking between her and her child.	0.87	0.07	0.82	0.03
**PACOTIS Overprotective Parenting**				
I insist upon always keeping my baby close to me, within my eyesight, and in the same room as I am.	0.71	0.07	0.68	0.07
I can never bring myself to leave my baby with a babysitter.	0.49	0.08	0.47	0.08
When I leave my baby with a babysitter, I miss him/her so much that I cannot enjoy myself.	0.62	0.07	0.63	0.07
I prefer that my baby sleeps in the same room as me at night.	0.52	0.08	0.55	0.07
I consider myself a ‘real mother hen’.	0.35	0.09	0.36	0.08
**PACOTIS Hostile Parenting**				
I have been angry with my baby when he/she was particularly fussy.	0.83	0.04	0.84	0.03
I have raised my voice with or shouted at my baby when he/she was particularly fussy.	0.84	0.04	0.82	0.04
I have spanked my baby when he/she was particularly fussy.	0.41	0.07	0.40	0.07
I have lost my temper when my baby was particularly fussy.	0.73	0.05	0.74	0.04
I have left my baby alone in his/her bedroom when he/she was particularly fussy.	0.23	0.08	0.23	0.08
I have shaken my baby when he/she was particularly fussy.	0.34	0.08	0.34	0.08
When my baby cries, he/she gets on my nerves.	0.84	0.91	0.47	0.07
**SDQ Conduct Problems**				
Often has temper tantrums or hot tempers.	0.58	0.10	0.61	0.08
Generally obedient.	0.46	0.09	0.48	0.08
Often argumentative with adults.	0.74	0.10	0.67	0.07
Can be spiteful to others.	0.32	0.10	0.38	0.09
